# The Influence of Chinese College Students’ Physical Exercise on Life Satisfaction: The Chain Mediation Effect of Core Self-evaluation and Positive Emotion

**DOI:** 10.3389/fpsyg.2021.763046

**Published:** 2021-11-23

**Authors:** Feiyang Liu, Zhengguang Zhu, Bo Jiang

**Affiliations:** ^1^Physical Education College, Southwest University, Chongqing, China; ^2^Faculty of Psychology, Southwest University, Chongqing, China; ^3^School Physical Education Development Institute, Southwest University, Chongqing, China

**Keywords:** physical exercise, core self-evaluation, positive emotion, life satisfaction, college students

## Abstract

Physical exercise is an important way for college students to maintain their physical health, and life satisfaction is one of the important indicators of college students’ mental health. Therefore, this study aims to explore the relationship between physical exercise and life satisfaction of college students. Additionally, we also seek to demonstrate the chain mediating effects of core self-evaluation and positive emotion on this relationship. A total of 794 Chinese college students, 324 men and 470 women, participated in the study. The participants were 17–25years old (M=19.96±1.54). They completed the Exercise Adherence Questionnaire, Core Self-evaluation Scale, Positive Affect and Negative Affect Scale, and Satisfaction with Life Scale. Results showed a strong positive relationship between physical exercise and life satisfaction and verified the mediating effect of core self-evaluation and positive emotion on this relationship. The results also confirmed the chain mediating model between physical exercise, core self-evaluation, positive emotion, and life satisfaction. It enlightens us that we should pay more attention to the organic combination of students’ physical activities and mental health education.

## Introduction

As the backbone of social development, college students’ physical and mental health is very important. In health studies, life satisfaction is an individual’s perception and evaluation of his or her overall life quality (Dew and Huebner, 1994), which is an important indicator to predict individual health level. Generally, individuals’ comprehensive health increases with the level of satisfaction that have toward their current life state (Chapman et al., 2019). Physical exercise is one of the main ways for college students to maintain their physical and mental health. It refers to the physical activities with certain intensity, frequency, and time that individuals carry out in their spare time for the main purpose of health (Song, 2001). Many studies have shown that physical exercise has a positive protective effect on the level of individual cardiopulmonary level, muscle tissue, and brain cognition, and has a good therapeutic effect on metabolic diseases (Sarmento et al., 2017; Leal et al., 2018; Hernandez et al., 2019; Borges et al., 2020). So does physical exercise affect life satisfaction? Although few studies have directly examined the relationship between physical exercise and life satisfaction, a number of studies have examined the positive effects of physical exercise on mental health. For example, moderate physical exercise can promote the improvement of individual psychological regulation ability, reduce the level of depression and anxiety, and can significantly improve the individual’s quality of life (Kvam et al., 2016; Frederiksen et al., 2021; Nguyen et al., 2021). When individuals are physically healthy, emotionally sound and have a high quality of life, they may also be more satisfied with their current life. In other words, physical activity may be an important factor in life satisfaction. Does physical activity affect life satisfaction through other factors?

We hypothesized that core self-evaluation may be an important mediator variable. Core self-evaluation is an integrated personality trait that includes self-esteem, control point, neuroticism, and general self-efficacy and constitutes a basic self-evaluation (Judge and Bono, 2001). For one thing, physical exercise may have an impact on core self-evaluation. A previous study conducted a self-report questionnaire survey on 326 adolescents and found that cultivating an active lifestyle through physical exercise promotes self-esteem more than reducing bad habits (Pazzaglia et al., 2020). Self-esteem is expressed by individuals through attitudes, language, and behaviors and through subjective evaluation of their own abilities, values, and meanings. Through physical exercise, college students can not only improve their physical fitness and enhance their satisfaction with their body self-image, but also gain friendship and gain recognition from others in the process of physical exercise, and then improve their self-esteem. And self-esteem is an important part of core self-evaluation, so physical exercise can also improve the level of core self-evaluation of college students (Ma and Liu, 2015). For another, core self-evaluation can have an impact on life satisfaction. For example, in a study of 319 students aged 17 to 21, the results showed that emotional intelligence and core self-evaluation explained 34% of the difference in life satisfaction, indicating that when adolescents’ core self-evaluation increased, their life satisfaction also increased (Esin et al., 2016). Other researchers verified that core self-evaluation is an important predictor of life satisfaction and further revealed its internal mechanism (Zhou and Xuan, 2015). In conclusion, both physical exercise and core self-evaluation can affect life satisfaction, while physical exercise can predict core self-evaluation. Therefore, core self-evaluation may play an important intermediary role between physical exercise and life satisfaction.

We hypothesized that positive emotion might be another important mediator. Positive emotion is short-term experiences that can cause changes in people’s thoughts, actions, and psychological reactions (Fredrickson and Branigan, 2005). Research on physical exercise and positive emotion shows that physical exercise not only helps improve individual muscle stiffness and promote metabolism but also can effectively relieve individual stress and increase individual positive emotional performance (Brett et al., 2016; Aguirre-Loaiza et al., 2019). In terms of the relationship between positive emotion and life satisfaction, researchers found that positive emotion had a significant predictive effect on life satisfaction, and school students with higher positive emotional experiences also experienced higher life satisfaction (Datu and King, 2016). Emotions are an indispensable part of an individual’s daily life experience. College students who often express positive emotional experiences will develop positive and optimistic attitudes. Even if they encounter negative life events, they are more likely to adopt positive coping methods to deal with problems, enhance the acceptability of negative events, and thus improve the level of life satisfaction (Jiang et al., 2021). It means that physical exercise can improve an individual’s life satisfaction by promoting the expression of positive emotion. Therefore, positive emotion may also be an important mediator between physical exercise and life satisfaction.

Although the above analysis shows that core self-evaluation and positive emotion may mediate the relationship between college students’ physical exercise and life satisfaction, this study holds that they do not simply play an independent mediating role but may also have a chain mediation effect. In the definition of core self-evaluation proposed by Judge, general self-efficacy is an important part of core self-evaluation. General self-efficacy consists of four aspects, namely, performance achievement, alternative experience, verbal persuasion, and physiological state (Bandura, 1977). In a study looking at what parents can do to promote healthy development in their children, it was found that when parents help children develop higher levels of general self-efficacy, children will show more positive emotion. Individuals’ general self-efficacy is an important cognitive resource. Individuals with low self-efficacy tend to overestimate the difficulty of the problem when completing tasks, which leads to anxiety and a higher level of psychological pressure and negative emotion. In contrast, when individuals with high self-efficacy face pressure due to highly challenging events, a high level of self-efficacy can stimulate their self-motivation to overcome difficulties, effectively help them relieve the negative impact caused by stress, and increase their positive and optimistic emotions. Fu and Tremayne found a significant relationship between healthy behaviors, self-efficacy, and positive emotion. The stronger the sense of self-efficacy, the more healthy behaviors and the more significant positive emotion expressed (Fu and Tremayne, 2021). Students with high self-efficacy generally exhibit more healthy behaviors, which is conducive to developing healthy living habits, promotes improvements in life quality, and thus is more conducive to positive emotional experiences (Hascher and Hagenauer, 2016). Therefore, we speculate that core self-assessment can influence life satisfaction through positive emotion.

In summary, this study constructed a chain mediation model between physical exercise and life satisfaction, in order to explore the relationship between physical exercise and life satisfaction and its internal mechanism. Thus, we hypothesized that as:

*Hypothesis 1*: Physical exercise would be positively associated with core self-evaluation, positive emotion, and life satisfaction.

*Hypothesis 2*: In the influence mechanism of college students’ physical exercise on life satisfaction, the core self-evaluation and positive emotion play a mediating role, respectively, and there is a chain mediating effect from core self-evaluation to positive emotion.

## Materials and Methods

### Participants and Procedures

Participants were undergraduate college students recruited from two colleges and universities (one key and one regular) in western China. We adopted a cluster random sampling method and selected 16 classes (two classes in each grade of two universities) to conduct a questionnaire survey. A total of 860 questionnaires were collected, and 794 valid questionnaires were left after excluding those that were not answered seriously or not standardized. Among them, 429 students are from key universities, with 130, 108, 101, and 90 students from first grade to senior grade, 140 boys, and 289 girls. The other students are from regular universities, with 105, 102, 82, and 76 students from first grade to senior grade, 184 boys, and 181 girls. All the students were aged from 17 to 25years old (M=19.96±1.54).

This study was approved by the Research Ethics Committee of Chinese Southwest University. Prior to the study, we contacted the administrators of the participating schools, obtained permission for the questionnaire test and the informed consent of the students themselves. All students participated in the survey voluntarily.

### Measures

#### Physical Exercise

Physical exercise was measured by the Exercise Adherence Questionnaire (Wang et al., 2016). This scale contains 14 items. It measures amateur physical exercise in three dimensions, including effort input (for example, “Regardless of whether I like physical exercise, I will do my best to complete it every time.”), emotional experience (for example, “I feel refreshed physically and mentally after exercise.”), and behavioral habits (for example, “I have been exercising regularly for at least 6months.”). The scale measures the individual’s actual exercise behavior and subjective experience after exercise and has good reliability and validity. Each item was rated on a 5-point scale (1=totally disagree and 5=totally agree), with higher scores indicating more adequate physical exercise. In this study, Cronbach’s α was 0.95 for the total scale and ranged from 0.87 to 0.91 for the subscales.

#### Core Self-evaluation

Core self-evaluation was measured by the Core Self-evaluation Scale (Judge and Bono, 2001). The scale has good reliability and validity under the Chinese cultural background (Gu and Wen, 2014). The Core Self-evaluation Scale includes 10 items covering four personality traits: self-esteem, control points, neuroticism, and general self-efficacy. A 4-point scale was used for scoring (completely disagree=1 and completely agree=4). The Cronbach’s α coefficient in this study was 0.85.

#### Positive Emotion

Positive emotion was measured by the positive affect and negative affect scale (PANAS) compiled by Watson (Watson et al., 1988). This scale contains a 10-item subscale of positive emotion and a 10-item subscale of negative emotion. Each item was rated on a 5-point scale (1=none of the time and 5=most of the time), with higher scores indicating more positive emotional experiences. The PANAS has been shown to have high reliability and validity in the Chinese school environment (Guo and Gan, 2010). The Cronbach’s α coefficient in this study was 0.90.

#### Life Satisfaction

Life satisfaction was measured by the Satisfaction with Life Scale compiled (Diener et al., 1985). Individuals evaluate their overall life satisfaction. This scale contains 5 items measured using a 7-point rating scale from 1 (completely nonconformance) to 7 (completely conforming), with higher scores indicating higher life satisfaction. This scale is suitable for use with adolescents in the Chinese school environment, and the reliability of the whole scale is excellent (Xiong and Xu, 2009). The Cronbach’s α coefficient in this study was 0.85.

#### Analytic Strategy

The study used SPSS 22.0 and the Process plug-in to analyze the data and used Process model 6 to test the chain mediation model. For the significance test of the regression coefficient, the bootstrapping method with 5,000 repeated samples was selected to obtain a robust standard error and a 95% deviation-corrected confidence interval (CI). When the CI does not contain zero, the effect is significant.

At the beginning of the data analysis, we used Harman’s single-factor test to explore whether there may be common method biases in this study. The results showed that there were seven factors with eigenvalues greater than 1; among these, the largest factor explained 32.26% of the variance, less than the critical range of 40%. Therefore, we believe that the possibility of common method biases in this study is relatively small.

## Results

### Descriptive Statistics and Analysis of the Correlations Between Variables

Descriptive statistics and correlation analysis were conducted for each variable. As shown in Table 1, there was a significant positive correlation between physical exercise, core self-evaluation, positive emotion, and life satisfaction.

**Table 1:** Descriptive statistics and interrelations among all observed variables.

Variables	*M*	SD	1	2	3	4
1 Physical exercise	49.78	11.66	–			
2 Core self-evaluation	33.49	6.20	0.25^***^	–		
3 Positive emotion	33.04	6.60	0.54^***^	0.41^***^	–	
4 Life satisfaction	22.27	5.47	0.35^***^	0.39^***^	0.54^***^	–

*** *p<0.001*.

### Testing the Mediating Effects of Core Self-evaluation and Positive Emotion

**Figure 1:**
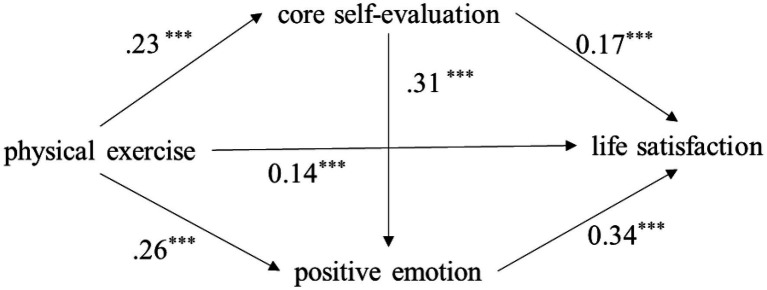
Model of the mediator role of core self-evaluation and positive emotion in the relationship between physical exercise and life satisfaction. *** p<0.001, Significant regression coefficient.

Taking physical exercise as the independent variable, life satisfaction as the dependent variable, and core self-evaluation and positive emotion as the mediating variables, the chain mediation effect test yielded the results shown in Figure 1 and Tables 2, 3.

**Table 2:** The chain mediation model from physical exercise to life satisfaction.

Variable	*b*				*c*				*d*			
	*β*	SE	*t*	95%CI	*β*	SE	*t*	95%CI	*β*	SE	*t*	95%CI
a	0.23	0.02	7.03^***^	[0.09, 0.17]	0.26	0.02	15.68^***^	[0.23, 0.30]	0.14	0.02	2.75^**^	[0.01, 0.08]
b					0.31	0.03	10.18^***^	[0.25, 0.38]	0.17	0.03	6.07^***^	[0.12, 0.23]
c									0.34	0.03	11.28^***^	[0.28, 0.40]
R^2^	0.16				0.37				0.33			
F	36.18^***^				118.01^***^				78.46^***^			

*** *p<0.001*.

*a, physical exercise; b, core self-evaluation; c, positive emotion; and d, life satisfaction, the same below*.

**Table 3:** Standardized indirect effects from physical exercise to life satisfaction.

Model	β (standardized indirect effect)	SE	95%CI	Relative mediating effect
Total indirect effect	0.147	0.013	0.10, 0.16	51.58%
a-b-d	0.039	0.006	0.01, 0.04	13.68%
a-c-d	0.084	0.013	0.06, 0.12	29.47%
a-b-c-d	0.024	0.003	0.01, 0.02	8.42%

The results show that, firstly, there are significant positive correlations between physical exercise, core self-evaluation, positive emotions, and life satisfaction, which supports Hypothesis 1. Secondly, physical exercise has a significant indirect effect on life satisfaction through core self-evaluation (*β*=0.039, 95% CI=[0.01, 0.04]), that is, core self-evaluation has a significant mediating effect on the relationship between physical exercise and life satisfaction. Thirdly, physical exercise has a significant indirect effect on life satisfaction through positive emotions (*β*=0.084, 95% CI=[0.06, 0.12]), that is, positive emotions have a significant mediating effect on the relationship between physical exercise and life satisfaction. Finally, the indirect effect of physical exercise on life satisfaction through core self-evaluation and positive emotion was significant (*β*=0.024, 95% CI=[0.01, 0.02]). In other words, core self-evaluation can affect positive emotion. At the same time, the chain mediation path from physical exercise to core self-evaluation, then to positive emotion, and finally to life satisfaction is significant, supporting Hypothesis 2. These results indicate that core self-evaluation and positive emotion play a continuous mediating role in the relationship between physical exercise and life satisfaction. The total mediating effect accounted for 51.58% of the total effect; that is, 51.58% of the effect of physical exercise on college students’ life satisfaction through the two variables of core self-evaluation and positive emotion.

## Discussion

As expected, physical exercise, core self-evaluation, positive emotion, and life satisfaction had significant positive relationships with each other. In addition, we found that core self-evaluation and positive emotion played a mediating role in the relationship between physical exercise and life satisfaction, and the chain mediation effect was significant.

First of all, from the relationship between physical exercise and core self-evaluation, physical exercise can significantly positively predict core self-evaluation. Although no studies have directly examined the relationship between physical exercise and core self-evaluation, existing studies have shown that physical exercise can significantly increase an individual’s self-esteem or self-awareness (Mcauley et al., 1997; Wong et al., 2021). In the process of physical exercise, the individual’s body will become stronger, which will improve the individual’s positive body self-image. At the same time, individuals can make friends with the same interests, which also provides them with peer support and promotes the improvement of self-esteem. Therefore, core self-evaluation, as a comprehensive reflection of self-esteem and self-awareness, will also be positively affected by physical exercise. Secondly, physical exercise significantly positively predicted positive emotion was also consistent with prior research (Greenwood, 2019). Physical exercise can promote the body to secrete dopamine, which is closely related to pleasure, thus increasing the individual’s positive emotional experience. Additionally, consistent with prior research, physical exercise was positively associated with life satisfaction (Moreno-murcia et al., 2017; Reigal et al., 2019). From the function of physical exercise, moderate physical exercise can enhance the physical quality of students, while maintaining good physical health is the desire of most people. Therefore, it is understandable that moderate physical exercise can improve college students’ life satisfaction. In conclusion, the results of this study demonstrate the positive promoting effect of physical exercise on mental health, and also support Hypothesis 1.

As for Hypothesis 2, physical exercise can directly affect life satisfaction and can also affect life satisfaction through the mediation of core self-evaluation and positive emotion. From the perspective of core self-evaluation, physical exercise can improve core self-evaluation. Individuals with higher core self-evaluation have stronger psychological adjustment ability, are more likely to use positive coping strategies, and have higher life satisfaction (Zhao and Shi, 2018). Therefore, core self-evaluation can play an important role in the bridge between physical and mental health, which is consistent with the existing findings (Xiang et al., 2019). From the perspective of positive emotion, physical exercise can also promote the production of individual positive emotion. When individuals experience more positive emotion, the proportion of negative emotions is significantly reduced, which helps individuals form positive and optimistic attitudes, have a greater sense of hope, and show higher life satisfaction (Ligeza et al., 2019; Bo et al., 2020). The results also showed that the mediation path of positive emotion has the greatest influence on the total mediation effect, indicating that the influence of physical exercise on life satisfaction is largely played by positive emotion. Moreover, we also found that core self-evaluation and positive emotions play a chain mediating role in the impact of physical exercise on college students’ life satisfaction. In other words, core self-evaluation affects life satisfaction by influencing positive emotion. Although there are relatively few studies that directly examine the relationship between core self-evaluation and positive emotions, many studies have found that the improvement of core self-evaluation will improve the level of self-esteem, enhance the experience of positive emotions, and adopt a more positive way to face difficulties and challenges, thus improving subjective wellbeing and work efficiency (Kim et al., 2016; Ding and Lin, 2020; Firouznia et al., 2021). This study directly proves that the improvement of core self-evaluation can enhance individuals’ positive emotional experience, and further proves the importance of core self-evaluation in individuals’ positive mental health.

In general, through physical exercise, individuals can improve their physical quality, gain friendship, enhance self-confidence, and thus improve their core self-evaluation. Meanwhile, in this process, individuals can enhance their positive emotional experience and ultimately improve their life satisfaction. This enlightens us, physical health and mental health are closely linked, we should encourage students to take an active part in physical exercise activities, cultivate a strong physique, promote healthy body, also need to guide students to establish positive self-evaluation, cultivate a positive, optimistic attitude toward life, and try to sports activities and the organic integration of mental health education.

## Limitations and Future Directions

This study also has some deficiencies. First, due to the limitation of objective factors, such as time and research funds, this study adopted a cross-sectional study design. Although existing studies have provided a solid foundation for this study, the results of this study can be enriched and expanded through further follow-up and empirical studies. Second, the subjects of this study were all college students without distinction. In future research, we can further explore the impact of physical exercise on life satisfaction of people of different ages. Third, physical exercise in this study was self-reported by participants through questionnaires. In the future, we can further explore the influence of frequency, time, and form of physical exercise on life satisfaction.

## Conclusion

In conclusion, this study investigates how college students’ physical exercise influences life satisfaction. Specifically, we found that physical exercise significantly positively predicted life satisfaction and verified the mediating role of core self-evaluation and positive emotions in this relationship. The results also confirmed a chain mediation model between physical exercise, core self-evaluation, positive emotions, and life satisfaction.

## Data Availability Statement

The raw data supporting the conclusion of this article will be made available by the authors, without undue reservation.

## Ethics Statement

The studies involving human participants were reviewed and approved by the Research Ethics Committee of Southwest University. Written informed consent to participate in this study was provided by the participants’ legal guardian/next of kin.

## Author Contributions

On the basis of reading related literature, FL raised the questions of this research and was responsible for making research plans, collecting data, and writing articles during the whole research process. ZZ made a great contribution to the data collection and analysis of the research and the revision of the paper. BJ was mainly responsible for the supervision and guidance of the whole research process and provided the necessary financial and personnel assistance. All authors contributed to the article and approved the submitted version.

## Funding

This research has been supported by the Key Project of Chinese Ministry of Education during the 14th Five-Year Plan Period (Title: Research on the bottleneck and collaborative governance of the construction of Chinese campus football characteristic schools in the new era; Serial number: DLA210371).

## Conflict of Interest

The authors declare that the research was conducted in the absence of any commercial or financial relationships that could be construed as a potential conflict of interest.

## Publisher’s Note

All claims expressed in this article are solely those of the authors and do not necessarily represent those of their affiliated organizations, or those of the publisher, the editors and the reviewers. Any product that may be evaluated in this article, or claim that may be made by its manufacturer, is not guaranteed or endorsed by the publisher.
